# Novel Antimicrobial Peptides from the Arctic Polychaeta *Nicomache minor* Provide New Molecular Insight into Biological Role of the BRICHOS Domain

**DOI:** 10.3390/md16110401

**Published:** 2018-10-23

**Authors:** Pavel V. Panteleev, Andrey V. Tsarev, Ilia A. Bolosov, Alexander S. Paramonov, Mariana B. Marggraf, Sergey V. Sychev, Zakhar O. Shenkarev, Tatiana V. Ovchinnikova

**Affiliations:** M.M. Shemyakin & Yu.A. Ovchinnikov Institute of Bioorganic Chemistry, the Russian Academy of Sciences, Miklukho-Maklaya str. 16/10, 117997 Moscow, Russia; 79175492709@yandex.ru (A.V.T.); b_off2@mail.ru (I.A.B.); apar@nmr.ru (A.S.P.); thpcb92@mail.ru (M.B.M.); svs@ibch.ru (S.V.S.); zakhar-shenkarev@yandex.ru (Z.O.S.); ovch@ibch.ru (T.V.O.)

**Keywords:** antimicrobial peptide, polychaeta, innate immunity, BRICHOS domain, recombinant peptide, α-helix, Rana-box, nuclear magnetic resonance (NMR)

## Abstract

Endogenous antimicrobial peptides (AMPs) are among the earliest molecular factors in the evolution of animal innate immunity. In this study, novel AMPs named nicomicins were identified in the small marine polychaeta *Nicomache minor* in the Maldanidae family. Full-length mRNA sequences encoded 239-residue prepropeptides consisting of a putative signal sequence region, the BRICHOS domain within an acidic proregion, and 33-residue mature cationic peptides. Nicomicin-1 was expressed in the bacterial system, and its spatial structure was analyzed by circular dichroism and nuclear magnetic resonance spectroscopy. Nicomicins are unique among polychaeta AMPs scaffolds, combining an amphipathic *N*-terminal α-helix and *C*-terminal extended part with a six-residue loop stabilized by a disulfide bridge. This structural arrangement resembles the Rana-box motif observed in the α-helical host-defense peptides isolated from frog skin. Nicomicin-1 exhibited strong in vitro antimicrobial activity against Gram-positive bacteria at submicromolar concentrations. The main mechanism of nicomicin-1 action is based on membrane damage but not on the inhibition of bacterial translation. The peptide possessed cytotoxicity against cancer and normal adherent cells as well as toward human erythrocytes.

## 1. Introduction

Endogenous antimicrobial peptides (AMPs), also known as host-defense peptides (HDPs), are among the most ancient molecular components of the innate immunity system that contribute to the first line of defense against pathogens of most life forms [1]. The complex membrane-targeting mechanism of their antimicrobial action and the ability to rapidly kill pathogens prevent the evolution of resistance to AMPs. Some AMPs inhibit a number of metabolic processes via interaction with intracellular targets [2]. Marine invertebrate animals have no acquired immunity and lack a system of antibody diversification. They live in a microbe-laden environment and use AMP-based defense against potential pathogens. Polychaeta is a largely unexplored class of invertebrates in the context of discovery of new AMPs. The large majority of polychaeta species are marine animals that inhabit all places, from the Arctic to the Antarctic, and from the littoral zone to the deepest depths of the oceans. They are considered the most primitive annelids, based on morphology, physiology, and development [3]. To date, AMPs have been identified in several species of polychaetes: 21-residue β-hairpin arenicins from *Arenicola marina* [4,5], 51-residue perinerin from *Perinereis aibuhitensis* [6], 22-residue α-helical hedistin from *Nereis diversicolor* [7], and 22-residue β-hairpin alvinellacin from *Alvinella pompejana* [8]. These AMPs are predominantly expressed in coelomocytes and therefore actively participate in cellular immunity. Notably, all the peptides were directly isolated from comparatively large polychaeta. Also, a putative β-hairpin antimicrobial peptide, designated capitellacin, was predicted based on the genome data of polychaeta *Capitella teleta* [8].

Here, we report novel AMPs, named nicomicin-1 and -2, found in the small polychaeta *Nicomache minor* (the Maldanidae family, Figure S1). The peptides were found under the project aimed at searching for novel AMPs from marine animals that dwell in the White Sea, a part of Arctic Ocean. *N. minor* is a benthic polychaeta widespread in the North Atlantic, North Pacific, and Arctic regions at depths of up to 100 m. *N. minor* permanently lives in cold water in massive hard tubes attached to stones [9], which is fundamentally different from the Arenicolidae and Capitellidae families that have borrowing and mobile lifestyles and thermotolerant *Alvinella pompejana* that dwells in active deep-sea hydrothermal vents. Nicomicin-1 has a unique spatial structure compared to other polychaeta AMPs. The peptide adopts an *N*-terminal α-helix and *C*-terminal extended part, bearing a loop stabilized by a disulfide bridge. Surprisingly, this structure resembles those of amphibian host-defense peptides having the Rana-box motif. The propiece of nicomicin precursor includes the BRICHOS domain, which is known to participate in the complex post-translational processing of proteins and possesses anti-amyloid chaperone activity [10]. The BRICHOS domain has been described in polychaeta only as precursor of β-hairpin AMPs. Therefore, the obtained results reveal that the BRICHOS domain could participate in the biosynthesis of different structural types of polychaeta AMPs. The identification procedure, structural organization of the precursor protein, spatial structure, biological activities, and structure-functional analysis of nicomicin-1 are described in this paper.

## 2. Results and Discussion

### 2.1. Nicomicin Is a Novel BRICHOS Domain-Related AMP

There are a number of methods for searching for novel AMPs: direct peptide isolation from tissues and cells, whole genome and/or transcriptome sequencing followed by bioinformatic analysis, cloning of cDNA amplified by primers targeting for conserved regions. The latter, in particular, is used to identify novel cathelicidins due to the high homology of cathelin-like domains (CLDs) among vertebrate species [11]. Direct peptide isolation of AMPs is a challenge due to the labor-consuming process of catching small solitary animals. A weak sequence homology between known polychaeta AMPs makes it difficult to identify novel peptides by sequence similarity-based methods. The BRICHOS domain is found in precursor proteins of β-hairpin antimicrobial peptides of polychaetes. So far, this domain has been reported for precursors of five AMPs: arenicin-1 and -2 [4], arenicin-3 also known as NZ17000 [12], alvinellacin [8], and capitellacin [8]. In contrast with the CLD, the sequence homology of the BRICHOS domain is quite low among polychaetes. The high variability of the BRICHOS domain sequences, even within a single species, may be associated with an adaptation mechanism enabling the correct biosynthesis of β-hairpin peptides in certain environments [13].

**Figure 1:**
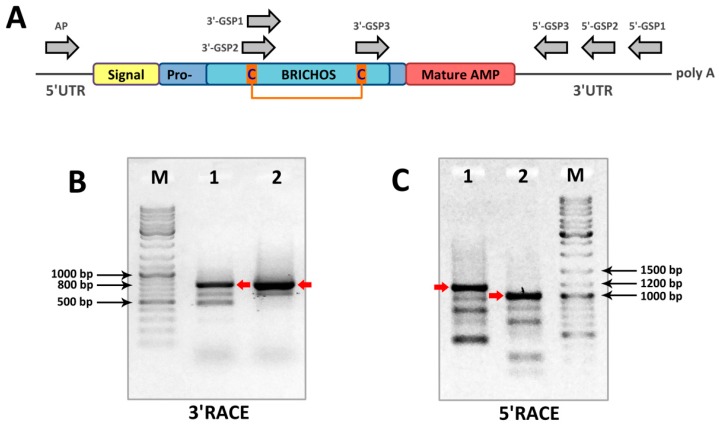
Rapid amplification of cDNA ends (RACE) analysis and identification of BRICHOS-related peptides in cDNA of *Nicomache minor*. (**A**) Scheme of prepronicomicin mRNA and primer annealing sites. Two rounds of step-out polymerase chain reaction (PCR) using nested gene-specific primers (GSP) were performed for both 3′RACE and 5′RACE. First round of 3′RACE was performed separately using 3′-GSP1 or 3′-GSP2 and step-out primer mix1. Then, diluted products were used in the second round with 3′-GSP3 and step-out primer mix2. The PCR products of the second round of 3′RACE were visualized by agarose gel electrophoresis: (**B**) M—marker; 1—3′-GSP1/3′-GSP3 product; and 2—3′-GSP2/3′-GSP3 product. The target bands are marked with red arrows. The first round of 5′RACE was performed with 5′-GSP1 and step-out primer mix1. Then diluted products were used in the second round using 5′-GSP2 or 5′-GSP3 and step-out primer mix2. The PCR products of the second round of 5′RACE were visualized by agarose gel electrophoresis: (**C**) M—marker; 1—5′-GSP1/5′-GSP2 product; 2—5′-GSP1/5′-GSP3 product. The one-round amplification of whole prepronicomicin coding sequence was then performed using AP and 5′-GSP2 primers.

In this study, the rapid amplification of cDNA ends (RACE) approach was implemented to identify novel BRICHOS-related peptides using *N. minor* cDNA and degenerate gene-specific primers (GSP, Table 1) that anneal to sequences with the highest primary structure homology among the BRICHOS domains of precursors of polychaeta AMPs (arenicin-1, arenicin-3, capitellacin, alvinellacin), specifically to sequences within two regions near conservative Cys residues (Figure 1A and Figure S2). One-round 3′RACE with any degenerate GSP and the universal adaptor-specific mix (step-out primer mix) failed to amplify fragments of interest. Therefore, two-round nested PCR was performed. Cloning and sequencing of 3′RACE products (Figure 1B), approximately 800 bp in length, revealed the sequence coding for the *C*-terminus of the BRICHOS domain, a putative mature AMP, and the 3′ untranslated region (3′UTR) of its cDNA. The 3′UTR length was found to be similar in several analyzed clones. Notably, two tandem repeats were found within the 3′UTR. This information was considered when designing 5′RACE GSPs. A 5′RACE (Figure 1C) using antisense GSPs annealing 3′UTR and adaptor primers provided sequence data extending to the 5′-end of the transcript.

**Figure 2:**
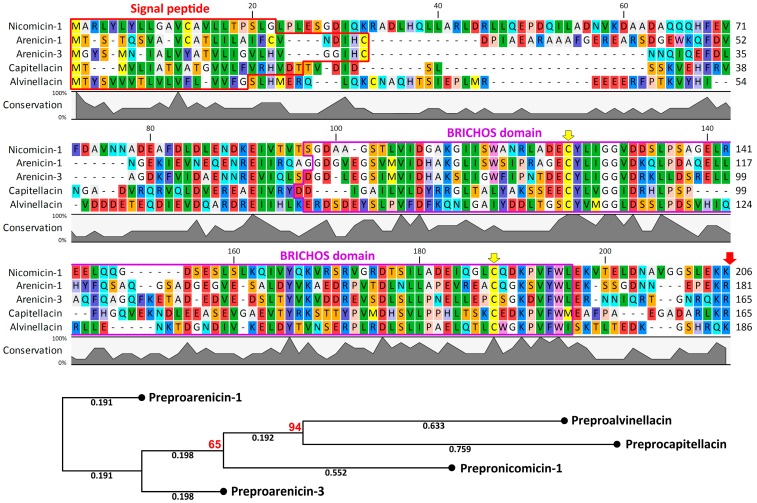
Amino acid sequence alignment and a neighbor-joining phylogenetic tree of the precursors without mature peptides of nicomicin-1, arenicin-1, arenicin-3, capitellacin, and alvinellacin. The alignment and phylogenetic tree were constructed using CLC Sequence Viewer software (version 8.0). Bootstrap values >50 are presented at the nodes and marked in red. The values were obtained from 1000 replicates. Signal peptide sequence identified with SignalP 4.1 (http://www.cbs.dtu.dk/services/SignalP/) and BRICHOS domain sequence identified with MyHits Motif Scan (https://myhits.isb-sib.ch/cgi-bin/motif_scan) are highlighted with red and purple boxes, respectively. The conservative cysteine residues in BRICHOS domain are marked with yellow arrows. Putative post-translational processing sites are marked with a red arrow.

The full-length mRNA sequence included a 720-bp open reading frame encoding a 239-residue prepropeptide consisting of a putative 22-residue signal sequence region, a proregion with the dibasic propeptide cleavage site, and the last 33 residues constituting the mature cationic peptide (Figure S3). Sequence analysis with the use of SignalP 4.1 (Figure 2) pointed out the Gly22-Leu23 bond as the most probable cleavage site for eukaryotic signal peptidase. The Glu-Lys-Lys motif preceding a putative propeptide cleavage site of nicomicin indicated that a precursor is probably activated by a subtilisin-like proprotein convertase [14]. More specifically, the kexin/furin family of proteases recognizes such dibasic sites—typically Arg-Arg or Lys-Arg and less frequently Arg-Lys, Lys-Lys, or Arg-X-X-Arg. This is a common feature for other BRICHOS-related AMPs (Figure 2). The Glu-Lys-Lys site was found to be a specific substrate for serine protease ASP, a putative virulence factor of the Gram-negative facultative anaerobic bacterium *Aeromonas sobria* [15]. This bacterium is known to cause infections of marine animals, and the nicomicin propeptide cleavage by proteases of invading pathogens (or epibionts) cannot be excluded.

**Figure 3:**
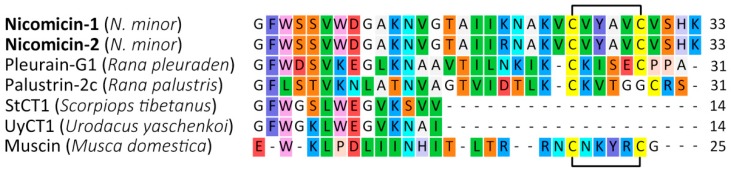
Amino acid sequence alignment of mature nicomicins with known antimicrobial peptides. The disulfide bonds are marked with square brackets.

A number of clones were analyzed after one-round amplification of the whole prepronicomicin nucleotide sequence with the use of 5′-GSP2 and the designed AP primer annealing to the 5′UTR. As the result, the 5′-terminal part of cDNA coding for the second isoform (named nicomicin-2) was found. This isoform has a single amino acid substitution, K19R. The precursor of nicomicin-2 bears three amino acid substitutions (V71E, D99G, and K191N), whereas the 5′UTR has a 6-bp deletion (GTTACA). At the same time, several different transcripts coding nicomicin-1 were identified, and polymorphisms were detected both in the signal sequence and in the propiece. The sequences coding prepronicomicins have been deposited in GenBank with the accession IDs MH898866–MH898867. Similar to known polychaeta AMPs arenicins, alvinellacin, and capitellacin, nicomicins are processed from a larger precursor molecule containing a signal peptide and an anionic proregion that includes the BRICHOS domain. This domain was found in many evolutionary distant animals and performs different functions [16,17]. It is characterized by low amino acid sequence conservation with only two Cys and one Asp residue, which are strictly conserved in all representatives of the BRICHOS superfamily [18]. The BRICHOS domain is known to be a part of proteins associated with different human diseases such as dementia, chondrosarcoma, and respiratory distress syndrome [19]. It participates in the complex post-translational processing of proteins and possesses anti-amyloid chaperone activity [10]. In this study, for the first time, the BRICHOS domain was shown to be a part of a precursor of AMP with a structure different from β-hairpin (Figure 3). Therefore, the BRICHOS domain might be a universal prodomain that participates in the biosynthesis of different types of AMPs in polychaeta, like the cathelin-like domain (CLD) does in vertebrates.

### 2.2. Nicomicin Is Unique Among Polychaeta AMPs but Shares Structural Similarities with Other Animal Host-Defense Peptides

Nicomicin-1 is composed of 33 amino acid residues including four basic Lys, one acidic Asp, and two Cys, forming a disulfide bridge (Figure 3). The nicomicin-1 amphipathic structure (see corresponding section below), as well as amino acid composition with a net positive charge and plenty of hydrophobic residues, suggest antimicrobial potential. A number of invertebrate AMPs containing a single disulfide bond are known: arenicin-1 and -2 from the lugworm *Arenicola marina* [4], thanatin from the spined soldier bug *Podisus maculiventris* [20], muscin from the house fly *Musca domestica* [21], and scarabaecin from the coconut rhinoceros beetle *Oryctes rhinoceros* [22]. Bioinformatic analysis revealed that the mature nicomicins do not share similarity higher than ~43% with any known AMPs listed in different AMP databases. The peptides exhibit the highest homology with amphibian AMPs (Figure 3), in particular, pleurain-G1 from skin secretions of the frog *Rana pleuraden* [23] and palustrin-2c from skin secretions of *Rana palustris* [24]. Despite the weak sequence homology, nicomicins and amphibian AMPs have similar structure organization, including an *N*-terminal amphipathic α-helix and a *C*-terminal extended region containing a disulfide-stabilized loop. This *C*-terminal, motif known as ‘Rana-box’ (6- or 7-residue loop), has been found in many amphibian AMP families: esculentins, gaegurins, ranalexins, and others [25]. At the same time, nicomicins have fundamentally different molecular organization of AMP precursors [23].

Several peptides of different origins are characterized by the presence of the *N*-terminal loop stabilized by a single disulfide bridge and followed by an α-helix: toxin Oxt 4a from the lynx spider *Oxyopes takobius* [26], bacterial pediocin-like antimicrobial peptides [27], and islet amyloid polypeptides (IAPP, also known as amylin) from vertebrates [28]. Aggregation of IAPP into amyloid fibrils in islets of Langerhans is associated with type 2 diabetes. Interaction of the peptide with the lipid membrane is of particular interest as it increases the rate of peptide aggregation, which can in turn result in membrane disruption [29]. Human IAPP has been shown to possess antibacterial activity with potency dependent on its aggregation states [30]. Notably, the BRICHOS domain of the precursor of the integral membrane protein 2B (Bri2), a transmembrane protein expressed in several peripheral tissues and in the brain, effectively inhibits human IAPP fibril formation in vitro [31]. Therefore, the nicomicin propiece containing the BRICHOS domain could be considered as a polypeptide-binding IAPP. Finally, the *N*-terminal part of nicomicin is significantly homologous with the short α-helical AMPs StCT1 [32] and UyCT1 [33] from venom of the scorpions *Scorpiops tibetanus* and *Urodacus yaschenkoi*, respectively (Figure 3).

### 2.3. Recombinant Expression and Purification of Nicomicin-1 and Its Fragments

**Figure 4:**
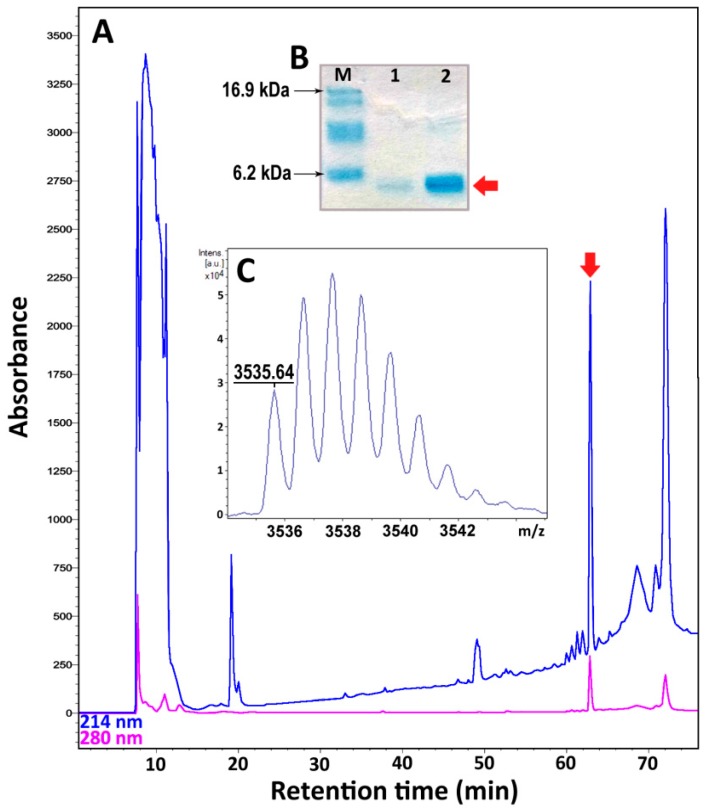
(**A**) Reverse-phase high-performance liquid chromatography (RP-HPLC) purification of the recombinant nicomicin-1. The fraction of recombinant nicomicin-1 is marked with a red arrow. (**B**) Tricine-SDS-polyacrylamide gel electrophoresis (Tricine-SDS-PAGE) of the recombinant nicomicin-1: M—molecular mass marker; 1—recombinant nicomicin-1 (3 µg); 2—recombinant nicomicin-1 (3 µg) boiled with 2-mercaptoethanol. (**C**) MALDI-TOF mass spectrometry analysis of the recombinant nicomicin-1. The experimental [M + H]^+^ monoisotopic mass is presented in the picture.

Nicomicin-1 is a hydrophobic peptide (51% of hydrophobic residues) stabilized by one disulfide bridge. Heterologous expression in the bacterial system of the peptide fused with a highly soluble carrier protein was applied for its production. To analyze the antimicrobial potential of the *N*-terminal amphipathic α-helix and the *C*-terminal extended region containing a disulfide-stabilized loop, the corresponding fragments of nicomicin-1, designated as Nico(1-17) and Nico(18-33), were obtained. In this study, modified thioredoxin A was used as a fusion partner that promoted the correct disulfide bond formation and masked the toxic effects of AMPs. Previously, several frog AMPs bearing Rana-box were obtained in the heterologous *Escherichia coli* expression system [34,35,36]. A high proportion of a fusion protein in reference to total cell protein was achieved with the use of thioredoxin A or glutathione S-transferase (GST). A final concentration of a fusion protein amounted to at least 1 mg/L. In this study, all the fusion proteins are expressed in *E. coli* BL21 (DE3) cells, and the obtained total cell lysates were fractionated by affinity chromatography. After purification and cleavage of the fusion proteins, reverse-phase high performance liquid chromatography (RP-HPLC) was used to obtain mature recombinant nicomicin-1 and its fragments (Figure 4A).

Tricine-SDS-polyacrylamide gel electrophoresis (Tricine-SDS-PAGE) revealed that the purified mature nicomicin-1 was expressed as a monomer (Figure 4B). Reduction of the disulfide bond with 2-mercaptoethanol did not alter electrophoretic mobility of the peptide but increased its interaction with Coomassie dye. The peptides were then analyzed by MALDI-TOF mass spectrometry. The calculated [M + H]^+^ monoisotopic molecular mass corresponding to the amino acid sequence of nicomicin-1 (3537.8 Da) exceeded the measured value (*m/z* 3535.6) by ~2 Da indicating formation of the disulfide bond between Cys24 and Cys29 and the absence of any other modifications (Figure 4C). The experimentally measured *m/z* values of the nicomicin-1 fragments matched the calculated molecular masses (Table 2). The final yields of the fragments were several-fold higher than for wild-type nicomicin-1 (0.9 mg per 1 L of the culture). Notably, induction of nicomicin-1 biosynthesis by isopropyl β-D-1-thiogalactopyranoside (IPTG) resulted in inhibition of *E. coli* BL21 (DE3) growth with a final optical density (OD_600_) value similar to that at the induction time point. In contrast, four hours after the fragments expression was IPTG-induced, the final OD_600_ increased at least three-fold, which is common for most AMPs according to our observations.

Both nicomicin-1 and its fragment Nico(1-17) were extremely hydrophobic, illustrated by the RP-HPLC retention times of 63 and 56 min, respectively. In comparison, the retention time of recombinant tachyplesin-1 (47% of hydrophobic residues) in the same system was 43 min. Both peptides were poorly soluble in water: nicomicin-1 formed visible aggregates at concentrations above 2 mg/mL, whereas Nico(1-17) formed a gel structure at a concentration of 2 mg/mL (Figure S4). Nicomicin-1 did not form oligomers on SDS-PAGE in contrast to arenicins [37]. Gel formation was reported for a range of classical amyloids or amyloid-like polypeptides, in particular for the above-mentioned IAPP [38]. In addition, the amyloid fibril-forming properties were shown for 17-residue α-helical AMP uperin 3.5 isolated from the skin secretions of the Australian toadlet *Uperoleia mjobergii* [39]. The presence of the *C*-terminal domain in the structure of nicomicin probably reduced the ability of the *N*-terminal α-helix to aggregate, but did not abolish it.

### 2.4. Nicomicin-1 Is Disordered in Aqueous Solution but Forms an α-Helical Structure in a Membrane-Mimicking Environment

**Figure 5:**
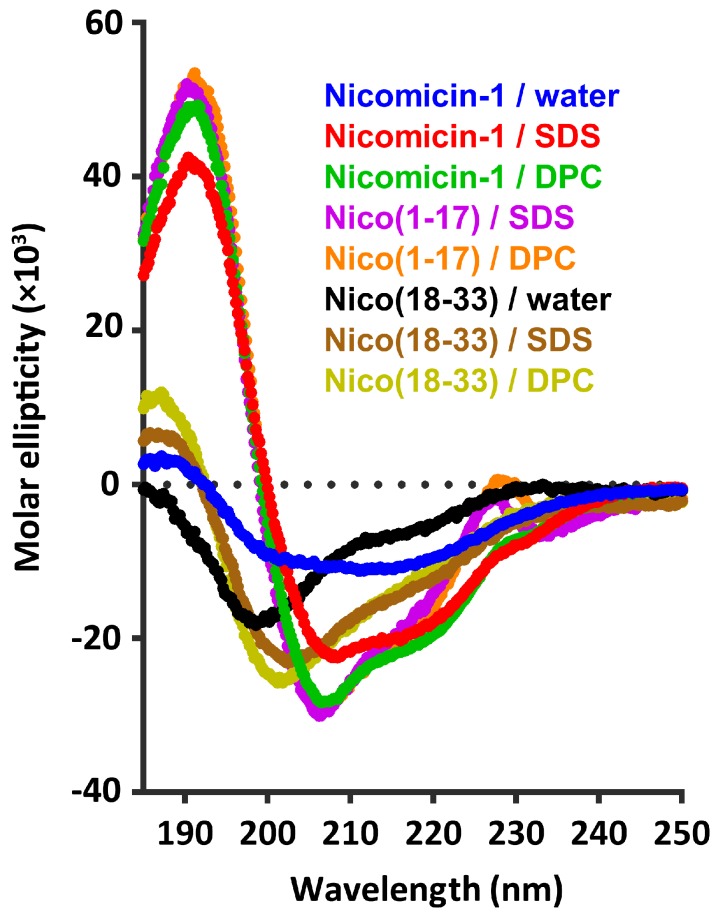
Circular dichroism (CD) spectra of nicomicin-1 and its fragments in water, 30 mM sodium dodecyl sulfate (SDS) micelles, and 30 mM dodecylphosphocholine (DPC) micelles. The spectrum of Nico(1-17) in water was not obtained in this study.

The structures of nicomicin-1 and its fragments were studied by circular dichroism (CD) spectroscopy. To observe the peptide structure changes upon interaction with lipid bilayers, we used anionic sodium dodecyl sulfate (SDS) and zwitterionic dodecylphosphocholine (DPC) micelles as a membrane-mimicking environment. Similar to lipid bilayers, the micelles have anisotropic properties, and hydrophobic regions of fatty tails are segregated from the polar water solution [40]. In contrast to isotropic mixtures of trifluoroethanol (TFE)/water or chloroform/methanol, the detergent micelles have a lower propensity to distort spatial structures of solubilized proteins or peptides and do not induce the formation of artificial helical structures [41]. As shown in Figure 5, the CD spectra of Nico(18-33) dissolved in water showed a negative peak at 200 nm, which indicated that the peptide mainly adopted a random coil conformation. The addition of micelles resulted in slight structuring of the peptide with the appearance of a weak positive peak at 190 nm. In contrast, the CD spectra of nicomicin-1 and Nico(1-17) in the membrane-mimicking environment showed a strong positive peak at 195 nm, and two negative peaks at 208 and 220 nm, which indicated that the peptides mainly adopted α-helix conformation.

**Figure 6:**
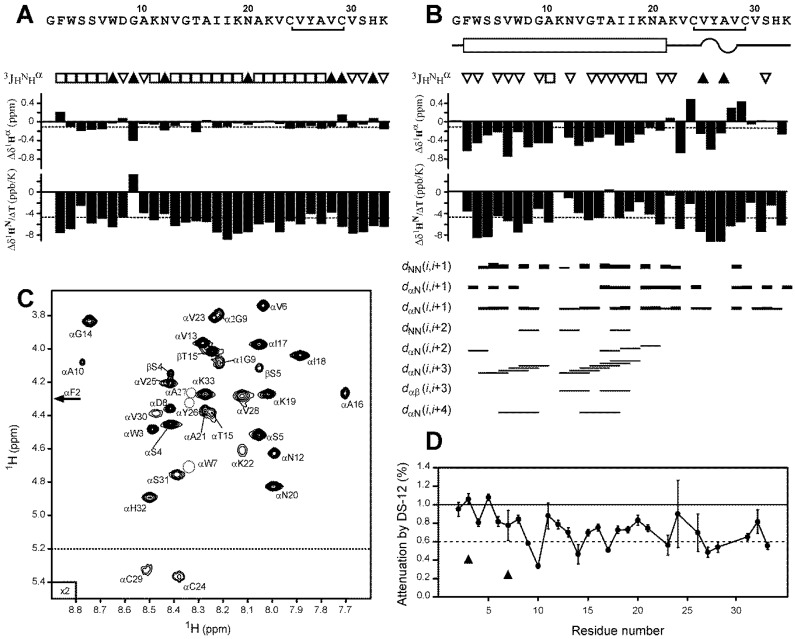
NMR data define the secondary structure of nicomicin-1 and topology of the peptide interaction with the dodecylphosphocholine (DPC) micelles. (**A**,**B**) Overview of NMR data collected for nicomicin-1 in H_2_O (pH 4.0, 30 °C) or DPC (pH 3.15, 45 °C, D:P ratio of 200:1) solutions, respectively. The resonances of Lys11 were not identified in the spectra and the residue remains unassigned. (From top to bottom) Secondary structure (helix—bar, β-turns—wavy line) of nicomicin-1 in the DPC micelles environment. Large (>8 Hz), small (<6 Hz), and medium (others) ^3^J_H_^N^_H_^α^ couplings are indicated by the filled triangles, open triangles, and open squares, respectively. The positive and negative values of the secondary ^1^H^α^ chemical shifts (Δδ^1^H^α^) correspond to β-structure and α-helix, respectively. The −0.1 ppm threshold value for helical secondary structure is shown by a dashed line. Amide protons demonstrating temperature gradients (Δδ^1^H^N^/ΔT) with amplitude <4.5 ppb/K could participate in hydrogen bond formation. NOE connectivities observed in the 100 ms two-dimensional (2D) NOESY spectrum for the peptide in DPC micelles are additionally shown in the panel (**B**). (**C**) The fragment of the 2D TOCSY spectrum of nicomicin-1/DPC sample (0.25/50 mM, pH 3.15, 45 °C). The resonance assignment is shown. The broadened signals that are below the drawing threshold are shown by dashed circles. (**D**) Attenuation of intensities of H^N^-H^α^ and H^N^-H^β^ cross-peaks in the 100 ms NOESY spectrum of the nicomicin-1/DPC sample by the paramagnetic probe (1 mM of lipid soluble 12-doxylstearate). The 0.6 threshold line subdivides data points in two groups: the residues situated inside or outside the micelle. The attenuation observed for ^1^H^ε^ resonances of the Trp3 and Trp7 side chains is shown by triangles.

For a detailed investigation of the nicomicin-1 structure, we employed the standard nuclear magnetic resonance (NMR) spectroscopy methods [42]. Complete ^1^H and partial ^13^C resonance assignments of the peptide in water were obtained at pH 4.0 and 30 °С. A summary of the obtained NMR data is shown in Figure 6A. The ^13^C^β^ chemical shifts of Cys24 and Cys29 residues were observed at 37.2 and 40.2 ppm, respectively, which are characteristic for oxidized Cys residues forming a disulfide bond. The small values of the secondary chemical shifts of ^1^H^α^ nuclei (−0.07 ppm on average) and lack of medium- and long-range nuclear Overhauser effect (NOE) contacts (data not shown) revealed the absence of defined secondary structure elements in the peptide molecule. This could be the consequence of the enhanced intramolecular mobility of nicomicin-1 in aqueous solution. The measured values of the ^3^J_H_^N^_H_^α^ coupling constants (~7 Hz, Figure 6A) are in agreement with the dynamic switching of the peptide backbone between α- and β-structural conformations. Thus, nicomicin-1 in a water environment adopts a disordered random structure.

It is generally assumed that micelles of anionic detergents, compared to zwitterionic ones, better mimic the negatively charged bacterial membranes. Some of the anionic detergents (e.g., SDS) are well known as harsh denaturing agent, that are able to disrupt tertiary and secondary structures of proteins. The CD data obtained in SDS and DPC solutions revealed that nicomicin-1 is less structured in the SDS micelles (Figure 5, red and green traces, respectively). Therefore, to investigate the spatial structure of nicomicin-1 in a membrane-mimicking environment, we used the zwitterionic DPC micelles solution. An extreme broadening of the nicomicin-1 resonances was observed upon titration of the peptide sample with DPC. The majority of cross-peaks in the amide region of two-dimensional (2D) Total Correlation Spectroscopy (TOCSY) and Nuclear Overhauser effect spectroscopy (NOESY) spectra were broadened beyond the detection limit at detergent to peptide molar ratios (D:P) from 5:1 to 75:1. Further increase in DPC concentration to D:P of 100:1 resulted in the narrowing of the amide proton signals, and cross-peaks in the 2D spectra became visible. The observed resonance broadening is the consequence of the peptide exchange between bulk aqueous phase and the micelles. To minimize the influence of this exchange process, we used a D:P ratio of 200:1 for the structural study. We assumed that at these conditions almost all peptide molecules were in the micelle-bound form. The conditions of NMR measurements were further optimized by varying sample pH and temperature. As a result, we obtained almost complete ^1^H and partial ^13^C resonance assignments of the peptide in DPC solution at pH 3.15 and 45 °С. Only one residue (Lys11) remained unassigned; its signals were not identified in the spectra. A summary of the measured NMR data is shown in Figure 6B.

Negative values of ^1^H^α^ secondary chemical shifts (−0.44 ppm on average), ^3^J_H_^N^_H_^α^ couplings with amplitude <6 Hz, and observed (*i*,*i* + 3) and (*i*,*i* + 4) H^α^-H^N^ NOE contacts (Figure 6B) revealed formation of an α-helix at the *N*-terminal region (Gly1-Ala21) of the peptide. Thus, interaction with the DPC micelles induces formation of a secondary structure in the nicomicin-1 molecule. At the same time, the temperature coefficients of the amide protons (Δδ^1^H^N^/ΔT) pointed to the low stability of this structure. Stable hydrogen bonds (characterized by |Δδ^1^H^N^/ΔT| < 4.5 ppb/K) were observed only on one side of the *N*-terminal helix. The ^1^H^N^ protons of Ser5, Gly9, Asn12, Val13, Ala16, and Ile18-Asn20 residues could participate in hydrogen bond formation. In the *C*-terminal part (Lys22-Lys33) of the peptide, only four stable hydrogen bonds can be formed. Together with the observed ^3^J_H_^N^_H_^α^ values, this indicates the absence of α-helix or β-structure in this region of the nicomicin-1 molecule.

Analysis of the fingerprint region in the 2D TOCSY spectrum (Figure 6C) revealed unequal line-widths and intensities of the ^1^H^N^ resonances. In the *N*-terminal fragment, significant broadening was observed for the amide proton of Trp7 and the resonances of Lys11 were probably broadened beyond the detection limit. Contrarily, 6 of 11 residues of the *C*-terminal region (Lys22, Cys24, Tyr26, Ala27, Cys29, and Val30) were significantly broadened. This indicated the presence of μs time-scale conformational exchange process(es), which are more pronounced in the *C*-terminal part of micelle-bound nicomicin-1.

**Figure 7:**
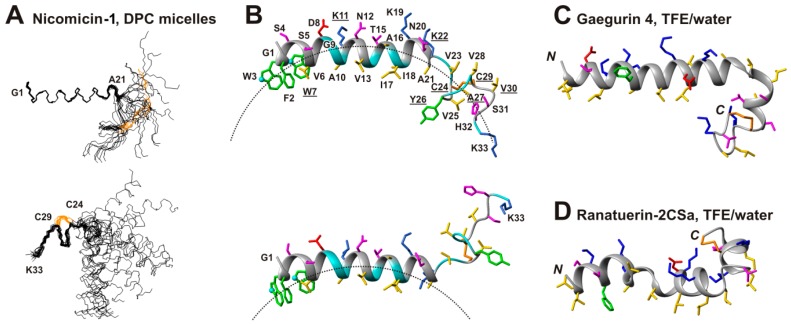
Spatial structure of nicomicin-1 in complex with dodecylphosphocholine (DPC) micelle. (**A**) The ensemble of 20 calculated nicomicin-1 structures is superimposed by the backbone atoms of *N*-terminal (Gly1-Ala21) and *C*-terminal (Lys22-Lys33) domains. The Cys24-Cys29 disulfide bond is shown in orange. (**B**) The representative conformers of nicomicin-1 in ribbon representation. The disulfide bond, positively charged, negatively charged, hydrophobic, aromatic, and polar residues are colored in orange, blue, red, yellow, green, and magenta, respectively. The approximate micelle surface (R ~24Å) is shown as a dashed line. The peptide ribbon is colored according to 12-doxylstearate paramagnetic relaxation enhancement data (presented on the Figure 6D). The residues located inside the micelle are colored in cyan. The N^ε^ atoms of the Trp3 and Trp7 side chains are shown by spheres. (**C**,**D**) Spatial structure of amphibian peptides gaegurin 4 and ranatuerin-2CSa, PDB codes 2G9L and 2K10, respectively.

The set of 20 nicomicin-1 structures (Figure 7A) was calculated in the CYANA program using experimentally derived distance and torsion angle restraints, and additional restraints that maintain closed backbone-backbone hydrogen bonds and disulfide (Table S1). We found that the peptide molecule consisted of two structurally independent domains connected by the hinge at the Lys22 residue. The *N*-terminal domain contains a prolonged α-helix (Phe2-Ala21) and its structure was precisely defined by NMR data (backbone root mean square deviation (RMSD) of 0.24 Å). In contrast, the *C*-terminal domain (Lys22-Lys33) adopts an extended conformation and accommodates two consecutive β-turns (Val25-Val30 residues) and a Cys24-Cys29 disulfide bond. Due to signal broadening and lack of experimental data, the structure of the *C*-terminal domain was less precisely defined (backbone RMSD of 0.69 Å).

### 2.5. Topology of Nicomicin-Micelle Interaction

The nicomicin-1 molecule in the DPC environment demonstrates a pronounced amphipathicity. The polar and charged residues are segregated at one face of the *N*-terminal α-helix and in the *C*-terminal Ser31-Lys33 region (Figure 7B). The other face of the α-helix and the region around the Cys24-Cys29 disulfide bond contains only aromatic and hydrophobic residues.

To elucidate the location of the nicomicin-1 molecule in the DPC micelle, we used a 12-doxylstearate relaxation probe. The nitroxide moiety of this probe is located in the hydrophobic region of the micelle [43]. Specific broadening of the nicomicin-1 proton signals was monitored using NOESY spectra at a DPC/probe molar ratio of 50:1 (i.e., about one relaxation probe per micelle). To characterize the effect of the relaxation probe on the nicomicin-1 protons, we compared the amplitudes of selected intraresidual cross-peaks with and without the probe (Figure 6D). The data indicated that two parts of the molecule were in contact with the hydrophobic region of the micelle. Significant cross-peak attenuation was observed for the residues located at the hydrophobic side of the α-helix in its central part (Gly9-Ile17), and in the region around the Cys24-Cys29 disulfide (Figure 6D). The *N*-terminal part of the α-helix (Gly1-Asp8) probably contacts the micelle only by aromatic side chain groups. Significant attenuation was observed for the signals of H^ε1^ protons of Trp3 and Trp7 side chains (Figure 6D, triangles).

The observed pattern of signal attenuation is consistent with the binding of the nicomicin-1 molecule to the surface of the DPC micelle by the *N*-terminal α-helix (Figure 7B). Due to significant scatter in the determined structures, the *C*-terminal domain could span different locations from being buried into the micelle to fully protruded into aqueous phase. Figure 7B illustrates this variability. The observed distribution of μs-timescale motions in the nicomicin-1 molecule (Figure 7B, underlined residues) indicated that the *C*-terminal domain underwent significant structural fluctuations. These fluctuations could be connected with the changes in the surrounding environment. We assume that the hinge-like motions around the Lys22 residue resulted in the dynamic partition of the *C*-terminal domain into the micelle. However, μs-timescale motions observed at Trp7-Lys11 residues forming one turn of the α-helix suggest the presence of another hinge region in the *N*-terminal domain.

To confirm incorporation of nicomicin-1 into DPC micelles, we measured the intrinsic fluorescence of Trp residues, which are known to be sensitive to the polarity of an environment [44]. Nicomicin-1 has two Trp residues (Trp3 and Trp7), and their fluorescence can be exploited to estimate the location of the peptide *N*-terminus in the micelle. A blue shift of emission maximum (353 → 332 nm) was observed upon transfer of nicomicin-1 from water to DPC micelles. This indicated that the Trp aromatic rings are in contact with the hydrophobic core of the micelle. The nicomicin-1 structure was also studied in water and in DPC micelles by fluorescence quenching experiments. Strict compliance with the Stern-Volmer equation was observed. Therefore, Trp3 and Trp7 are equally accessible for the quencher. Surprisingly, the transfer of nicomicin-1 from water to micelles only slightly decreased the Stern-Volmer constant from 9.2 to 8.5 M^−1^. The Trp side chains in the micelle-bound peptide probably experience an intensive dynamic (collisional) quenching. This may be caused by the presence of a positively charged group, which increases the concentration of the negative iodide (I^−^) quencher in the vicinity of Trp side chain within ~10 Ǻ [44,45]. According to the determined spatial structure of nicomicin-1 (Figure 7B), presumably the *N*-terminal amino group (NH_3_^+^-) is responsible for the observed effect. In addition, the quencher can effectively reach the Trp side chains, which may prove that they are located at the interface between the polar and hydrophobic region of the micelle. This location agrees well with the NMR data and proposed model of nicomicin-1/micelle complex (Figure 7B).

### 2.6. Comparison of Spacial Structures of Nicomicin-1 and Amphibian Peptides Containing ‘Rana-Box’ Motif

The α-helix in the nicomicin-1 molecule is terminated by the fragment containing several β-turns stabilized by a Cys24-Cys29 disulfide bond. This structural arrangement resembles the Rana-box motif found in helical antimicrobial peptides isolated from frog skin [25]. The spatial structures of several peptides from this family were previously determined by NMR spectroscopy, but only two structures (gaegurin 4 [46] and ranatuerin-2CSa [47]) are available in the PDB database (Figure 7C,D). Similar to nicomicin-1, both peptides contain prolonged amphipathic helical regions, whereas the *C*-termini of the molecules are capped by positively charged disulfide-stabilized Rana-box motifs. In contrast to amphibian peptides, the Rana-box in nicomicin-1 does not contain positively charged residues and demonstrates significant hydrophobicity. Notably, nicomicin-1 possesses the additional four-residue fragment after the Rana-box. This region of the molecule is relatively polar and contains basic Lys33 residue.

### 2.7. Antibacterial Activity and Mechanism of Action

The antibacterial activity of nicomicin-1 and its fragments was determined using a two-fold serial dilution assay in lysogeny broth (LB) medium with or without NaCl. Minimum inhibitory concentrations (MICs) of the peptides against Gram-positive and Gram-negative bacteria are presented in Table 3. Melittin, known as a potent cytolytic and bactericidal agent, was used as a positive control. Nicomicin-1 exhibited a pronounced antimicrobial effect against Gram-positive bacteria, similar to that of α-helical cationic peptide melittin. The activity against Gram-negative microorganisms was modest and at least two- to four-fold lower than that of melittin. Interestingly, the α-helix peptide hedistin from *Nereis diversicolor* was also inactive against a set of Gram-negative microorganisms, except the marine bacterium *Vibrio alginolyticus*, which is a causative agent of infections of marine invertebrates [7]. Surprisingly, none of the peptides inhibited growth of *Bacillus megaterium* at concentrations up to 16 µM. Previous structure–activity relationship (SAR) studies of Rana-box amphibian AMPs revealed that the cyclic *C*-terminal part of these peptides does not influence the antibacterial activity, whereas the *N*-terminal α-helix is responsible for membrane disruption [25]. The α-helical peptide Nico(1-17) did not show any antibacterial activity at concentrations of 16 µM or higher. Notably, the net charge of the entire Nico(1-17) was zero and its most homologous AMP, 14-residue StCT1 from scorpion *Scorpiops tibetanus*, displayed poor antibacterial properties [32]. Weak activity of Nico(18-33) was detected only against several Gram-positive bacteria in a salt-free medium.

**Figure 8:**
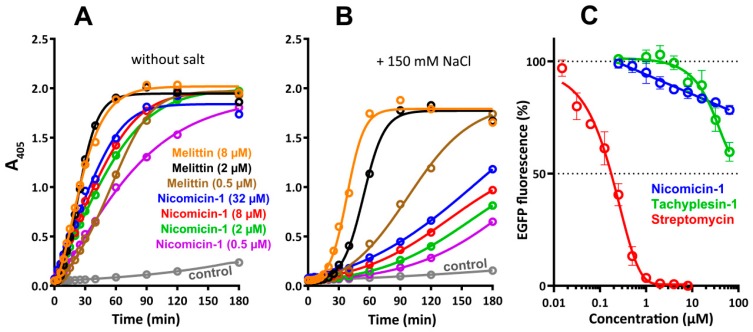
Analysis of nicomicin-1 antibacterial mechanism of action. *Escherichia coli* ML-35p cytoplasmic membrane permeabilization by nicomicin-1 at various concentrations from 0.5 to 32 µM, highlighted with colors (**A**) in the absence or (**B**) in the presence of NaCl. The cytolytic peptide melittin was used as the positive control from 0.5 to 8 µM, highlighted with colors. Three independent experiments were performed, and the curve pattern was similar for all three series. (**C**) Effects of nicomicin-1, tachyplesin-1, and streptomycin on the fluorescence resulting from the in vitro translation of EGFP using *E. coli* BL21 (DE3) Star cell extract. The data are presented as the mean ± SD of three independent experiments.

Cationic AMPs realize their antibacterial function by damaging membrane integrity and/or specifically inhibiting intracellular processes [2]. In this study, an effect of the peptides on bacterial cytoplasmic membrane integrity was analyzed with the use of *E. coli* ML-35p, a strain lacking the functional lactose permease necessary for the uptake of o-nitrophenyl-β-d-galactoside (ONPG) and constitutively expressing β-galactosidase. The latter produces o-nitrophenol, a chromogenic product with absorbance at 405 nm. Both nicomicin-1 and melittin effectively damaged membranes in a salt-free environment (Figure 8A). However, the addition of 150 mM NaCl markedly reduced the activity of nicomicin-1 (Figure 8B), which could explain the weak antibacterial effect of the peptide against *E. coli* in the presence of salt (Table 3). Salt probably both promotes peptide aggregation in a test medium and affects primary electrostatic attraction, thus impairing nicomicin-1 interaction with the bacterial membrane. This salt inhibiting effect is quite surprising in light of the marine origin of the host. Notably, both fragments of nicomicin-1 had a negligible ability to damage the membrane integrity of *E. coli* ML-35p, which was comparable to that of the negative control (data not shown). Therefore, the α-helical amphipathic part of nicomicin-1 cannot effectively interact with the membrane bilayer without the *C*-terminal fragment of the peptide and vice versa.

On the other hand, the ability of nicomicin-1 to inhibit its own biosynthesis in the *E. coli* expression system suggests a more complicated mechanism of action of the peptide against Gram-negative bacteria, which may differ from direct membrane destruction. Induction of nicomicin-1 biosynthesis resulted in inhibition of *E. coli* BL21 (DE3) growth (Section 2.3). Similarly, Pro-rich ribosome-targeting AMP apidaecin fused to a large carrier protein effectively inhibited growth of bacterial cells during heterologous expression in *E. coli* [48]. Therefore, we tested an ability of nicomicin-1 and other antimicrobial compounds to inhibit protein biosynthesis in vitro. The obtained results indicated a slight inhibition of the biosynthesis process. Nicomicin-1 caused only a 20% inhibition of enhanced green fluorescent protein (EGFP) expression at a concentration of 64 µM (Figure 8C). The observed effect was similar neither to streptomycin—a specific ribosome-targeting inhibitor of bacterial translation (IC_50_ 0.2 µM)—nor to tachyplesin-1 that effectively binds nucleic acids [49]. Therefore, the major mechanism of nicomicin-1 antibacterial action did not seem to be related to the inhibition of bacterial translation.

The question about the localization of the peptide in host tissues remains open. Polychaeta species are strictly dependent on epithelial barrier continuity and efficacy of the innate immune system, which are vitally important for their protection against pathogenic microorganisms. The BRICHOS-related peptides, arenicins and alvinellacin, are constitutively expressed both in coelomocytes and in the tegument of hosts, thus indicating involvement of the peptides in both systemic and local epithelial immunity [8,50]. Localization of the nicomicins needs further elucidation with the use of immunohistochemistry approaches.

### 2.8. Nicomicin-1 Possesses Cytotoxicity Against Mammalian Cells

**Figure 9:**
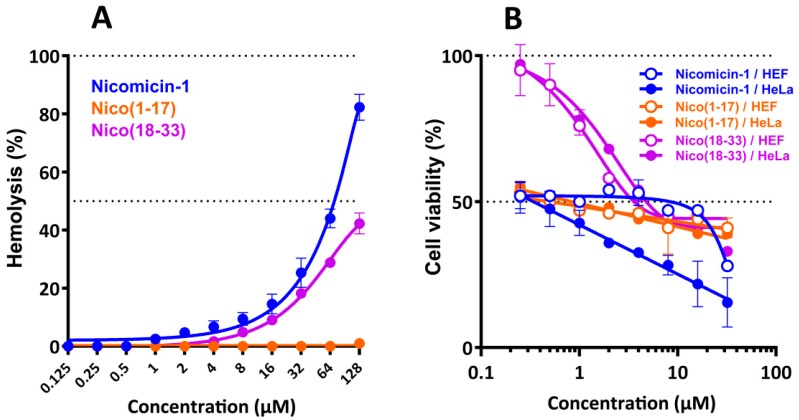
(**A**) Hemolytic activity of nicomicin-1 and its fragments after 1.5 h incubation (hemoglobin release assay). (**B**) Cytotoxicity of nicomicin-1 and its fragments against human embryonic fibroblasts (HEF) and cervix adenocarcinoma cells (HeLa) after 24 h incubation (3-(4,5-dimethylthiazol-2-yl)-2,5-diphenyltetrazolium bromide (MTT) dye reduction assay). The data are presented as the mean ± SD of three independent experiments.

Considering the ability of nicomicin-1 and Nico(1-17) to aggregate and potentially form fibrils in aqueous solutions, we analyzed the cytotoxic effects of nicomicin-1 and its fragments against adherent cell lines of human embryonic fibroblasts (HEF) and cervix adenocarcinoma cells (HeLa) as well as toward human red blood cells (hRBC). Nicomicin-1 and Nico(18-33) had similar hemolysis profiles with a half maximal hemolysis concentration (HC_50_) of about 64 and 128 µM, respectively (Figure 9A). In contrast, Nico(1-17) almost lacked hemolytic activity and lysed only 1% of red blood cells at a concentration of 128 µM. This is in agreement with data on antibacterial activity of Nico(1-17). Therefore, the *C*-terminal part of nicomicin-1 plays a key role in its hemolyticity. Cytotoxicity of nicomicin-1 toward HeLa cells was shown to be dose-dependent and attained a value of 85% of dead cells at the peptide concentration of 32 µM (Figure 9B). Nicomicin-1 possesses selectivity toward cancer cells. In contrast to hemolytic and antibacterial activities, the peptide fragment Nico(1-17) was cytotoxic toward adherent mammalian cells. Notably, the peptide Nico(1-17) showed a dose-independent cytostatic effect against both HeLa and HEF cells at a concentration range from 0.25 to 32 μM. The molecular mechanism of nicomicin-1 or Nico(1-17) cytotoxicity at submicromolar concentrations is still obscure. The above-mentioned amyloid fibril-forming amphibian AMP uperin 3.5 possessed a similar cytotoxic activity profile against pheochromocytoma cells [39]. Gel formation by amyloids within or outside cells as well as peptides adsorption could perturb membrane integrity, interfere with cell motility and signaling pathways, and alter the collagen gel network of the extracellular matrix [38]. Therefore, the activity of nicomicin-1 may also result from peptide aggregation.

## 3. Materials and Methods

### 3.1. Animal Collection

*Nicomache minor* (Arwidsson, 1906) tubeworms (Figure S1) were collected in June near the Nikolai Pertsov White Sea Biological Station (WSBS, Republic of Karelia, Russia) in the White Sea at depths of 5–35 m on silty sediments with stones using an ocean 0.1 m^2^ grab and diving technique. Several specimens of *N. minor* were cleaned with distilled water, cut, submerged in RNAlater solution (Thermo Fisher Scientific, Waltham, MA, USA) to preserve RNA before total RNA isolation, and stored at −20 °C until used.

### 3.2. Total RNA Isolation, RT-PCR, RACE Amplification

The tissue samples were thawed, removed from the solution, and homogenized in liquid nitrogen. Intact total RNA was isolated by using SV Total RNA Isolation System (Promega, Fitchburg, WI, USA). The RNA was quantified at 260 nm using a Ultrospec 3300 Pro spectrophotometer (Amersham Biosciences, Amersham, UK), and reverse transcribed into cDNA using a Mint RACE cDNA amplification kit (Evrogen, Moscow, Russia) according to manufacturer’s protocols. The obtained cDNA was analyzed by PCR amplification, cloning, and sequencing of the Folmer fragment of cytochrome oxidase subunit I (COI) gene. COI is adopted as the standard ‘taxon barcode’ for most animal groups [51]. Amplification of the 517-bp fragment was performed with two designed gene-specific primers: 5′-GGCACCTCTATAAGACTCCT-3′ and 5′-GAACTGGGAGGGAGAGAAGAA-3′. The analyzed sequence was shown to be identical to that of coding COI from *N. minor* isolate SPM11 (GenBank accession ID MG975588.1).

To determine the sequence of the 3′ and 5′ ends of cDNA coding a putative BRICHOS-related peptide, the RACE strategy was used. Two rounds of step-out PCR with the use of nested gene-specific primers (Table 1) were performed for both 3′ and 5′RACE (Figure 1). The 3′RACE was performed using degenerate GSPs that anneal to sequences encoding the two most conservative protein regions (Figure S2) in the BRICHOS domains of the precursors of polychaeta AMPs (arenicin-1, arenicin-3, capitellacin, and alvinellacin). The first round of 3′RACE was performed separately using 3′-GSP1 (outer) or 3′-GSP2 (outer) and adaptor primers—step-out primer mix1 supplied in the kit. Then 1000-fold diluted products were used in the second round with 3′-GSP3 (inner) and step-out primer mix2. Both rounds of nested PCR were performed using the same step-down technique with the following parameters: 95 °C for 60 s, 30 cycles of 94 °C for 30 s, annealing at 66→55 °C for 40 s (66-2, 62-3, 59-4, 57-5, 55-16 cycles) and extension at 72 °C for 60 s, and a final elongation step at 72 °C for 10 min. 5′RACE was performed as a nested PCR with GSPs complementary to the 3′UTR. The first round of 5′RACE was performed with 5′-GSP1 (outer) and step-out primer mix1. Then, 1000-fold diluted products were used in the second round using 5′-GSP2 (inner) or 5′-GSP3 (inner) and step-out primer mix2. Both rounds were performed using the same technique with the following parameters: 95 °C for 60 s; 30 cycles of 94 °C for 30 s, annealing at 62→55 °C for 40 s (62-3, 59-3, 57-4, 55-20 cycles) and extension at 72 °C for 90 s; and a final elongation step at 72 °C for 10 min. The one-round amplification of the whole coding sequence was performed using AP primer complementary to the 5′UTR and 5′-GSP2 with the following parameters: 95 °C for 60 s; 40 cycles of 94 °C for 30 s, annealing at 64→56 °C for 40 s (64-2, 62-3, 60-4, 58-5, 55-26 cycles) and extension at 72 °C for 60 s; and a final elongation step at 72 °C for 10 min. The products were separated by electrophoresis on 1.5% agarose gel and visualized on an ultraviolet (UV) transilluminator. The PCR products were purified from agarose gel and inserted into a pGEM-T vector (Promega, Fitchburg, WI, USA). The ligation products were transformed into the chemically competent *E. coli* DH10B cells. Plasmid DNA was isolated from white colonies on LB agar plates supplemented with ampicillin (100 µg/mL) using Plasmid Miniprep kit (Evrogen, Moscow, Russia). The plasmids were sequenced on both strands using the ABI PRISM 3100-Avant automatic sequencer (Applied Biosystems, Foster City, CA, USA).

### 3.3. Expression and Purification of the Antimicrobial Peptides

The recombinant plasmids for expression of nicomicin-1, as well as its fragments Nico(1-17) and Nico(18-33), were constructed with the use of pET-based vector as described previously [52]. The expression cassette included the T7 promoter, ribosome binding site (RBS), and the sequence encoding the recombinant protein that included an 8× His tag, the carrier protein (*E. coli* thioredoxin A with the Met37Leu mutation), a methionine residue, and a target peptide. *E. coli* BL21 (DE3) cells were transformed with the constructed plasmids and grown up to OD_600_ 1.0–1.5 at 37 °C in rich medium containing tryptone (15 g/L), yeast extract (17.5 g/L), NaCl (10 g/L), 20 mM glucose, 1 mM MgSO_4_, 0.1 mM CaCl_2_, 0.01 mM FeCl_3_, and 100 μg/mL of ampicillin, and then were induced with IPTG at a final concentration of 0.4 mM. The induction was performed at 30 °C for 4 h under shaking culture conditions at a speed of 220 rpm. The cells were sonicated in the 100 mM phosphate buffer (pH 7.8) containing 20 mM imidazole and 6 M guanidine hydrochloride to fully solubilize the fusion protein. Purification of the peptides involved immobilized metal affinity chromatography (IMAC) of cell lysate with the use of Ni Sepharose (GE Healthcare, Chicago, IL, USA), CNBr cleavage of the fusion protein, and RP-HPLC as described previously [53]. The recombinant peptides were characterized by Tricine-SDS-PAGE according to [54] and MALDI-MS (Bruker Daltonics, Bremen, Germany). Melittin and tachyplesin-1 used in this study were obtained as described earlier [52,55]. The peptides concentrations were estimated using UV absorbance.

### 3.4. Circular Dichroism Spectroscopy

Secondary structures of the peptides were analyzed by circular dichroism (CD) spectroscopy with the use of Jasco J-810 instrument (Jasco, Tokyo, Japan). Experiments were performed at 25 °C in water, in 30 mM DPC (Anatrace, Maumee, OH, USA) micelles, and in 30 mM SDS (Sigma, St. Louis, MO, USA) micelles. Final concentrations of peptides were of 150 μM. Four consecutive scans were performed and averaged, followed by subtraction of the blank spectrum of the solvent.

### 3.5. NMR Spectroscopy

For the NMR study in water and in DPC micelles environment, 0.4 and 0.25 mM samples of the recombinant nicomicin-1 were used, respectively. d38-DPC (CIL, Andover, MA, USA) was added to the NM sample in water using concentrated stock solution. The final DPC concentration was 50 mM. The pH of the samples were adjusted to 4.0 (water) or 3.15 (DPC) using concentrated HCl or NaOH solutions and 5% D_2_O was added. NMR spectra were measured at 30 °C (water) or at 45 °C (DPC) on an AVANCE-III 600 spectrometer (Bruker, Karlsruhe, Germany) equipped with cryogenically cooled probe (Bruker, Karlsruhe, Germany). 12-doxylstearate (Sigma, St. Louis, MO, USA) was added to the nicomicin-1/DPC sample (0.25/50 mM) using lyophilized aliquots of stock solution in methanol. NOESY spectra (τ_m_ = 100 ms) were measured at zero and 1 mM 12-doxylstearate concentrations at pH 3.15.

The protein resonance assignment was performed using a standard approach [42] using a combination of 2D ^1^H-TOCSY (τ_m_ = 80 ms), ^1^H-NOESY (τ_m_ = 60, 80 and 100 ms), and ^13^C-Heteronuclear Single Quantum Coherence spectroscopy (HSQC) spectra in the CARA (version 1.84, Zurich, Switzerland) program. The ^3^J_H_^N^_H_^α^ coupling constants were determined from line shape analysis of NOESY and TOCSY cross peaks in the Mathematica program (version 8.0, Wolfram Research, Champaign, IL, USA). The ^3^J_H_^α^_H_^β^ coupling constants were estimated from the multiplet patterns in 2D TOCSY spectrum. The spatial structure calculations were performed in the CYANA (version 3.97) program [56]. Upper interproton distance constraints were derived from NOESY cross-peaks (τ_m_ = 100 ms) via a “1/r^6^” calibration. Torsion angle restraints and stereospecific assignments were obtained from J coupling constants and NOE intensities. Hydrogen bonds were introduced using temperature coefficients of amide protons (Δδ^1^H^N^/ΔT) measured in the range of 20 to 45 °C in the TOCSY or NOESY spectra. Additional upper/lower restraints were applied to close the Cys24-Cys29 disulfide and backbone-backbone hydrogen bonds.

### 3.6. PDB and BMRB Accession Codes

The ^1^H and ^13^C chemical shifts of nicomicin-1 in H_2_O and DPC were deposited into the BioMagnetic Resonance Bank (BMRB, www.bmrb.wisc.edu, accession code 27611 and 34313, respectively). NMR constraints and derived atomic coordinates (20 models) for nicomicin-1 in complex with DPC micelle were deposited into the RCSB Protein Data Bank (PDB, https://www.rcsb.org, accession code 6HN9). Before PDB deposition, the obtained nicomicin-1 structure was checked by the internal PDB validation tool. Validation revealed good quality of the obtained structure with 81% of residues located in favored regions and 10% of residues in allowed regions on the Ramachandran plot (Table S1). The full report is available on the PDB website.

### 3.7. Tryptophan Fluorescence and Quenching

Tryptophan fluorescence was measured by means of Hitachi F-4000 (Hitachi, Tokyo, Japan) fluorescence spectrophotometer using 1 × 0.4 cm quartz cuvettes (Hellma Analytics, Müllheim/Baden, Germany). Emission and excitation slits were 5 nm wide. The excitation wavelength was 280 nm. Peptide concentration was 10 μM. The DPC to peptide molar ratio was of 200:1. The pH of the samples was of 4.0. Fluorescence was quenched by addition of increasing amounts of 4 M potassium iodide.

### 3.8. Antimicrobial Assay

The bacteria *Bacillus subtilis* B-886, *Micrococcus luteus* B-1314, and *Bacillus megaterium* VKM41 were obtained from All-Russian Collection of Microorganisms (Pushchino, Russia). *Rhodococcus* sp. SS1 was obtained from Institute of Biochemistry and Physiology of Microorganisms (Pushchino, Russia). *Bacillus licheniformis* VK21 was obtained from the Branch of M.M. Shemyakin & Yu.A. Ovchinnikov Institute of Bioorganic Chemistry (Pushchino, Russia). The extensively drug resistant clinical isolate of *Acinetobacter baumanii* was obtained from Sechenov First Moscow State Medical University hospital. Other strains were obtained from ATCC (Manassas, VA, USA). Bacterial test cultures were grown in LB medium (10 g/L tryptone, 5 g/L yeast extract, 10 g/L NaCl) at 37 °C to mid-log phase and then diluted with the same medium containing or lacking NaCl to reach a final cell concentration of 2 × 10^5^ CFU/mL. Then, 50 µL of the obtained bacterial suspension were added to 50 µL aquilots of the peptide solutions serially diluted with sterilized 0.1% bovine serum albumin (BSA) in 96-well flat-bottom polystyrene microplates (Eppendorf #0030730011, Hamburg, Germany). To avoid the adsorption of AMPs to plastic surfaces, an addition of BSA was used [57]. After incubation for 20 h at 30 °C and 900 rpm on a plate thermoshaker (Biosan, Riga, Latvia), the minimum inhibitory concentrations (MICs) were determined as the lowest peptide concentrations that prevented growth of test microorganisms observed as visible turbidity. In most cases, no significant divergence of MIC values was observed (within ±1 dilution step). The results are expressed as the median values determined on the basis of at least three independent experiments performed in triplicate.

### 3.9. Bacterial Membranes Permeability Assay

To examine an ability of the peptides to permeabilize the cytoplasmic bacterial membrane, a colorimetric assay with o-nitrophenyl-β-d-galactoside (ONPG, AppliChem, Darmstadt, Germany) and *E. coli* ML-35p strain was performed as previously described [58] with some modifications. The final concentration of ONPG and *E. coli* ML-35p cells were 2.5 mM and 2 × 10^7^ CFU/mL, respectively. Peptide samples were placed in a 96-well plate with a non-binding surface (NBS, Corning #3641, Corning, NY, USA), and an optical density of the solution was measured at 405 nm using the Multiskan EX microplate reader (Thermo Fisher Scientific, Waltham, MA, USA). The assay was performed in 10 mM sodium phosphate buffer with or without 150 mM NaCl at 30 °C under stirring at 300 rpm. Control experiments were performed under the same conditions without the addition of a peptide. Three independent experiments were performed, and the curve patterns were similar for all three series.

### 3.10. Cell-Free Protein Expression Assay

The cell lysate required for the translation inhibition assay was prepared using *E. coli* BL21 Star (DE3) cell culture grown at 30 °C in 2× YTPG liquid medium (1.6% tryptone, 1% yeast extract, 0.5% NaCl, 22 mM NaH_2_PO_4_, 40 mM Na_2_HPO_4_, 0.1 M glucose). The chromosome of DE3 strains contains a gene encoding T7 RNA polymerase under control of the lacUV5 promoter. The bacterial culture was grown to OD_600_ 0.8–1.0, then the T7 RNA polymerase gene was induced by addition of 0.2 mM IPTG. Bacteria were harvested at OD_600_ 5.0–6.0 by centrifugation (3000× *g*, 30 min, 4 °C). The bacterial pellet was washed three times by suspending it in four volumes of wash buffer (10 mM tris-acetate buffer, pH 8.2, 14 mM magnesium acetate, 60 mM potassium glutamate, and 1 mM dithiothreitol), then resuspended in one volume of the same buffer (1 mL per 1 g of wet cell mass) and disrupted by sonication at 5–15 °C. The total cell lysate was centrifuged at 15,000× *g* (30 min, 4 °C). The supernatant was split into aliquots and stored at −70 °C. In order to investigate effects of AMPs on the translation process, the peptides were added to a cell-free protein synthesis (CFPS) reaction mix with a plasmid encoding EGFP variant (F64L, S65T, Q80R, F99S, M153T, V163A) under a control of the T7 promoter. The reaction mix consisted of the following components: 1.2 mM ATP, 0.8 mM UTP, 0.8 mM GTP, 0.8 mM CTP, 2 mM of each of 20 proteinogenic amino acids, 1.5 mM spermidine, 1 mM putrescine dihydrochloride, 0.06647 mM calcium folinate, 170 ng/mL tRNA from the *E. coli* MRE 600 strain, 0.33 mM nicotinamide adenine dinucleotide (NAD), 10 mM ammonium glutamate, 175 mM potassium glutamate, 60 mM glucose, 120 mM HEPES-KOH (pH 8.0), 15 mM magnesium glutamate, 2% PEG 8000, 25% *E. coli* BL21 Star (DE3) cell lysate, and 10 ng/μL plasmid DNA. The reaction volume was 50 μL. The peptides were dissolved in water with the addition of 0.1% BSA. Streptomycin was used in the positive control reactions. Fluorescence of the sample without inhibitor was set to 100%. The reaction proceeded for 60 min in a 96-well clear flat-bottom black polystyrene microplates (Corning #3340, Corning, NY, USA) in a plate shaker (30 °C, 900 rpm). Fluorescence of the synthesized EGFP was measured with a AF2200 microplate reader (Eppendorf, Hamburg, Germany) (λ_Exc_ = 488 nm, λ_Em_ = 510 nm). The experimental data were obtained from at least three independent experiments.

### 3.11. Hemolysis and Cytotoxicity Assay

The hemolytic activity of the peptides was tested against the fresh suspension of human red blood cells (hRBC) using the hemoglobin release assay as described previously [53]. Three experiments were performed with hRBC from blood samples of independent donors. The quantitative data are represented as average means with standard deviations. The colorimetric 3-(4,5-dimethylthiazol-2-yl)-2,5-diphenyltetrazolium bromide (MTT) dye reduction assay was used to determine the cytotoxicity of the peptides against HeLa (cervix adenocarcinoma cells) and human embryonic fibroblasts (HEF) cell lines as described previously [52,59]. The experimental data were obtained from at least three independent experiments.

## 4. Conclusions

This study extends the knowledge of the structure and functions of AMPs from marine invertebrates, and in particular from the small polychaeta tubeworm *N. minor*. Overall, nicomicins represent a novel scaffold among polychaeta, AMPs combining an amphipathic *N*-terminal α-helix and *C*-terminal extended part with a six-residue loop stabilized by a disulfide bridge. The recombinant nicomicin-1 is structured in a membrane-mimicking environment, exhibits membrane-active properties, and possesses a pronounced activity against Gram-positive bacteria and cancer cells. The peptide shares similarities in both primary and secondary structure with amphibian host-defense peptides, and has a fundamentally different molecular organization of the precursor. The obtained results reveal that the BRICHOS domain does not exclusively participate in biosynthesis of β-hairpin polychaeta AMPs, but could also be a part of precursor of α-helical AMPs, namely nicomicins. Until now, this domain has been described in polychaeta only as a precursor of β-hairpin AMPs. Therefore, the BRICHOS domain might be a universal prodomain that participates in the biosynthesis of different structural types of AMPs in polychaeta by analogy with the cathelin-like domain in vertebrates.

## Figures and Tables

**Table 1:** Oligonucleotide primer sequences.

Primer Name	Sequence 5′→3′
3′-GSP1	GTGTTACGTCATGGGTGG(G,C)(G,C)T(G,T)GAC
3′-GSP2	GAGTGCTAC(T,C)TG(A,G)TCGG(A,C)GG
3′-GSP3	TGC(G,T,C)AGGG(A,C)AA(A,G)CCTGT(C,T)TTCTGG(A,C)T
5′-GSP1	GTGGTCAATGAATATCTGCAATACA
5′-GSP2	GAGCTTATACCCATAGGGCTTCCTTATAC
5′-GSP3	ATTAAGAACGTTGTCCAAAGCGTAATG
AP	GTTGATCCGACAGTCGCTTGC

**Table 2:** Properties of nicomicin-1 and its fragments.

Peptide	RP-HPLC Retention Time ^1^ (min)	Calculated [M + H]^+^ Monoisotopic Mass (Da)	Measured Monoisotopic *m*/*z* Value ^2^	Recombinant Peptide Final Yield (mg/L)
Nicomicin-1	63	3535.81 *	3535.64	0.9
Nico(1-17)	56	1794.87	1794.84	6.2
Nico(18-33)	42.5	1759.94 *	1759.97	4.3

^1^ Retention times of the peptides on semi-preparative reverse-phase high performance liquid chromatography (HPLC); ^2^ Molecular masses were determined using MALDI-TOF MS; * assuming two Cys residues form cystine.

**Table 3:** Antibacterial activity of the peptides.

Bacteria	Minimum Inhibitory Concentration (µM)							
	Melittin		Nicomicin-1		Nico(1-17)		Nico(18-33)	
	Without Salt	+NaCl	Without Salt	+NaCl	Without Salt	+NaCl	Without Salt	+NaCl
**Gram-positive**								
*Micrococcus luteus*	0.25	0.25	0.125	0.25	>16	>16	16	>16
*Bacillus subtilis*	0.5	0.5	0.062	0.25	>16	>16	16	>16
*B. licheniformis*	0.25	0.25	0.125	0.25	>16	>16	8	>128
*B. megaterium*	>16	>16	>16	>16	>16	>16	>16	>16
*Staphylococcus aureus* 209P	2	32	2	32	>16	>16	>16	>16
*S. aureus* ATCC 29213	1	1	2	16	>16	>16	>128	>128
*Rhodococcus sp.*	0.5	0.25	0.125	0.25	>16	>16	>16	>16
**Gram-negative**								
*E. coli* BL21 (DE3)	2	4	2	32	>64	>64	>64	>64
*E. coli* ML-35p	8	16	16	>32	>64	>64	>64	>64
*E. coli* C600	4	8	32	>32	>64	>64	>64	>64
*Acinetobacter baumanii*	8	32	32	>32	>64	>64	>64	>64
*Pseudomonas aeruginosa* PAO1	>32	32	32	>32	>64	>64	>128	>128

## Supplementary Materials

The following are available online at http://www.mdpi.com/1660-3397/16/11/401/s1. Figure S1: Polychaeta Nicomache minor; Figure S2: Design of degenerate gene-specific primers for amplification of the 3′-end of cDNA encoding BRICHOS-related peptides; Figure S3: The nucleotide sequence of mRNA encoding prepronicomicin-1 and its translation; Figure S4: The peptide Nico(1-17) forms gel structure at concentration of 2 mg/mL in water; Table S1: Statistics for the best CYANA structures of nicomicin-1 in DPC solution at pH 3.15.
