# Supplementary material for: Novel Antimicrobial Peptides from the Arctic Polychaeta Nicomache minor Provide New Molecular Insight into Biological Role of the BRICHOS Domain
Source: Mar Drugs. 2018 Oct 23;16(11):401. doi: 10.3390/md16110401 (PMC6265681; doi:10.3390/md16110401)
Supplement: Supplementary file 1 [file marinedrugs-16-00401-s001.pdf]

Supplementary material

Novel antimicrobial peptides from the Arctic polychaeta *Nicomache minor* provide new molecular insight into biological role of the BRICHOS domain

Pavel V. Panteleev *, Andrey V. Tsarev, Ilia A. Bolosov, Alexander S. Paramonov, Mariana B. Marggraf, Sergey V. Sychev, Zakhar O. Shenkarev, Tatiana V. Ovchinnikova

M.M. Shemyakin & Yu.A. Ovchinnikov Institute of Bioorganic Chemistry, the Russian Academy of Sciences, Miklukho-Maklaya str. 16/10, Moscow, 117997

* Correspondence: alarm14@gmail.com; ibch@inbox.ru; Tel.: +7-495-335-0900

Table S1. Statistics for the best CYANA structures of nicomicin-1 in DPC solution at pH 3.15

**Distance and Angle restraints**	
Total NOE contacts	260
intraresidual	77
sequential (|i-j|=1)	85
medium-range (1<|i-j|≤4)	98
Hydrogen bonds restraints (11 bonds, upper/lower)	22/22
S-S bond restraints (1 bond, upper/lower)	3/3
Torsion angle restraints	
Angle φ	26
Angle χ1	4
**Total restraints/per residue:**	340/10
**Statistics for calculated structures**	
Structures calculated/selected	200/20
CYANA target function ($Å^2$)	1.64 ± 0.1
Violations of restraints	
Distance (>0.2 Å)	4
Distance (>0.5 Å)	0
Dihedral angles (>5 °)	0
RMSD (Å) overall (Gly1-K33)	
Backbone	2.72 ± 0.80
All heavy atoms	3.42 ± 1.04
RMSD (Å) N-terminal domain (Gly1-Asn21)	
Backbone	0.24 ± 0.06
All heavy atoms	0.72 ± 0.09
RMSD (Å) C-terminal domain (Lys22-Lys33)	
Backbone	0.54 ± 0.32
All heavy atoms	1.33 ± 0.43
Ramachandran analysis	
Residues in favored regions (%)	81
Residues in allowed regions (%)	91

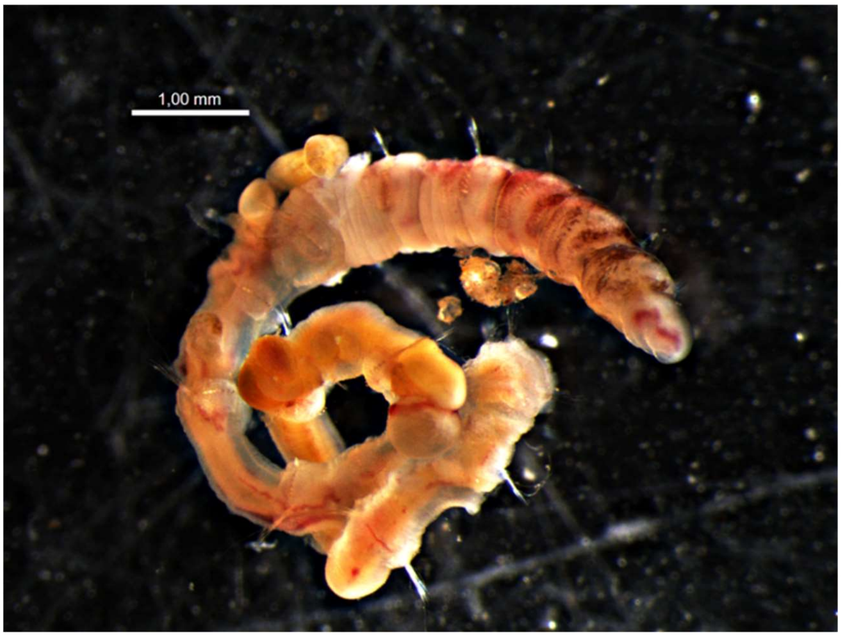

Figure S1. Polychaeta *Nicomache minor*

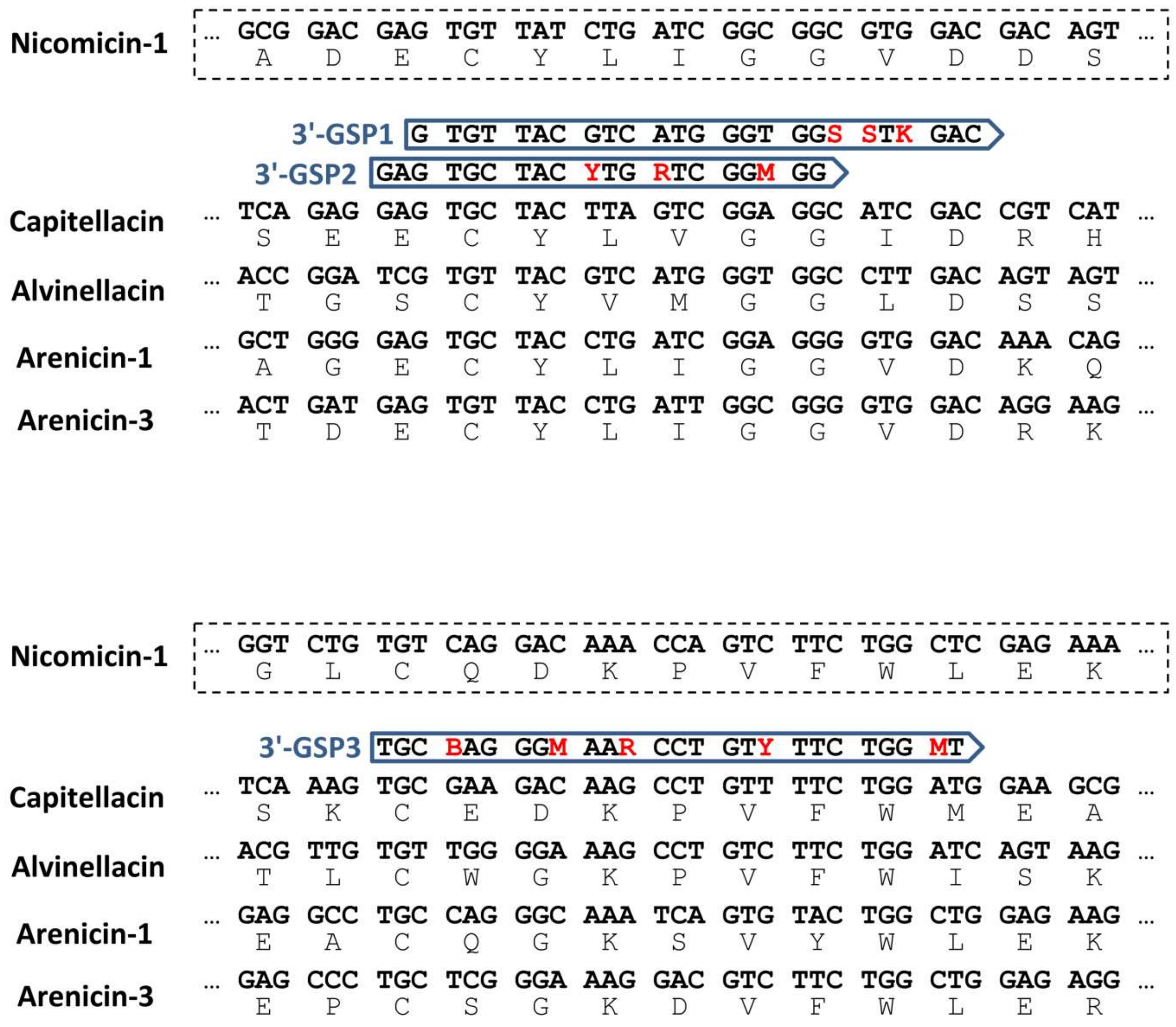

Figure S2. Design of degenerate gene-specific primers for amplification of the 3′-end of cDNA encoding BRICHOS-related peptides. Nucleotide and amino acid sequence alignments are presented at the figure. The primers were designed to anneal to sequences encoding two most conservative regions in BRICHOS domains of precursors of polychaeta AMPs capitellacin [8], alvinellacin [8], arenicin-1 [4], and arenicin-3 [12]. Degenerate nucleotides (R(A,G); Y(C,T); M(A,C); K(G,T); S(G,C); B(G,T,C)) in the primers are marked with red. 3′-GSP1 was designed to preferentially anneal to proalvinellacin encoding sequence. 3′-GSP2 was designed to preferentially anneal to procapitellacin and proarenicins encoding sequences. 3′-GSP3 was designed to preferentially anneal to consensus sequence (proarenicin-1, proarenicin-3, procapitellacin, and proalvinellacin). The corresponding pronicomicin-1 cDNA fragments are marked with dashed boxes.

GTTGATCCGACAGTCGCTTGCAAACGAACTCAGAAAAGTTACAGTGATGGCCCGACTATAT
M A R L Y

CTGTACCTGTTGGGAGCGGTGTGTGCCGTCCTGCTCACTCCCAGCCTGGGGCTGCCCCTTGAGTCCGGC
L Y L L G A V C A V L L T P S L G L P L E S G

GACATTCAGAAACGTGCCGACCTCCACCAGCTATTGGCTAGGCTGGACAGACTGCTCCAAGAACCGGAT
D I Q K R A D L H Q L L A R L D R L L Q E P D

CAGATACTTGCAGACAATGTCAAGGACGCTGCAGATGCTCAGCAACAGCATTTTGAGGTATTTGATGCT
Q I L A D N V K D A A D A Q Q Q H F E V F D A

GTGAATAACGCAGACGAAGCATTCGACCTTGACCTTGAGAATGACAAGGAAATCGTTACAGTGACCAGT
V N N A D E A F D L D L E N D K E I V T V T S

GGTGACGCCGCCGGGTCTACCCTTGTCATAGATGGAGCCAAGGGTATCATCAGCTGGGCCAACAGGCTG
G D A A G S T L V I D G A K G I I S W A N R L

GCGGACGAGTGTTATCTGATCGGCGGCGTGGACGACAGTCTGCCCTCTGCAGGGGAACTCCGGGAGGAA
A D E C Y L I G G V D D S L P S A G E L R E E

CTGCAGCAGGGAGACTCTGAGTCGTTGTCGCTGAAGCAGATCGTGTACCAGAAGGTCCGTAGCCGAGTG
L Q Q G D S E S L S L K Q I V Y Q K V R S R V

GGCCGGGACACGTCTATCTTGGCAGACGAGATCCAAGGTCTGTGTCAGGACAAACCAGTCTTCTGGCTC
G R D T S I L A D E I Q G L C Q D K P V F W L

GAGAAAGTCACGGAACTGGACAATGCCGTTGGAGGATCTTTGGAGAAGAAGGGTTTTTGGAGCAGTGTT
E K V T E L D N A V G G S L E K K G F W S S V

TGGGACGGGGCGAAGAATGTGGGGACGGCAATCATCAAAAATGCCAAAGTTTGCGTATACGCGGTATGC
W D G A K N V G T A I I K N A K V C V Y A V C

GTCAGCCACAAGTGATCTCCCCACGACCAGCAAGAAGCGAAATGGACATTCTTGGATATATATGTTCCT
V S H K .

GATTTTGATTGAAACAAACTAATTTAAACTTCGCTTAGATACCTTATAATATGTCTGGTAAAACTGCTC

CTAAGATAATATTATAACAATGTTCATTACGCTTTGGACAACGTTCTTAATAACATGATTGATTCAGGT

GAACATTTATATGCTTCGCGCGCGATGTTGCGACCCTTACAAAACAAATGATGCTTACATAAAAGAAAT

AACATGATTGCATTCAGAACGGCAACATTTAGTATAAGGAAGCCCTATGGGTATAAGCTCCATTTGCCT

ATTGATTTTAAATACAATGCTGTTGACAAATGTAATAAGGTTTATATAAATTAATGGTTCATTTTTACC

AGTCTGATATTACAAATAGATATCATGTATTGCAGATATTCATTGACCACATAAAATCAATTACTTACT

TAATTTCTTGCTTTCATAATTTCATATAATAAGTTGAAATAAAACGTTTAGCTTCAAAAAAAAAAAAAA

AAAAAAAAAAAAAAAA

Figure S3. The nucleotide sequence of mRNA encoding prepronicomicin-1 and its translation. The open reading frame is represented with the following individual components: signal peptide (brown), prosequence (blue); and mature peptide (red). Tandem repeat sequences at the 3′UTR are underlined.

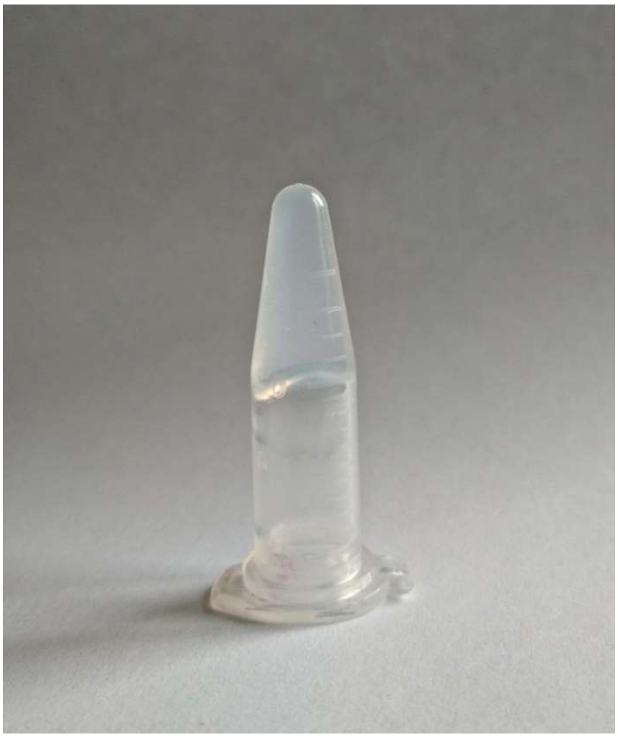

Figure S4. The peptide Nico(1-17) forms a gel structure at concentration of 2 mg/mL in water.
